# Resection of corticotroph microadenomas invading the medial wall of the cavernous sinus in two cases of a primary and recurrent case of Cushing’s disease

**DOI:** 10.3171/2023.4.FOCVID2323

**Published:** 2023-07-01

**Authors:** Ahmed Mohyeldin, Maximiliano Alberto Nunez, Yuanzhi Xu, Juan C. Fernandez-Miranda

**Affiliations:** 1 Department of Neurosurgery, Stanford University Medical Center, Stanford, California;; 2 Department of Neurosurgery, University of California, Irvine, Orange, California;; 3 Department of Neurosurgery, Shanghai General Hospital, Shanghai Jiao Tong University School of Medicine, Shanghai, China

**Keywords:** Cushing’s disease, pituitary adenoma, cavernous sinus invasion, medial wall of the cavernous sinus, biochemical remission

## Abstract

Emerging evidence from multiple highly specialized groups continues to support a role for resection of the medial wall of the cavernous sinus when it is invaded by functional pituitary adenomas, to offer durable biochemical remission. The authors present two cases of Cushing’s disease that underscore the power of this surgical technique in achieving remission in microadenomas that ectopically present in the cavernous sinus or have invaded the medial wall of the sinus. This video demonstrates key steps in the safe removal of the medial wall of the cavernous sinus and successful resection of tumor burden in the cavernous sinus for sustained postoperative remission.

The video can be found here: https://stream.cadmore.media/r10.3171/2023.4.FOCVID2323

## Transcript

### 0:21 Review of the Literature and Relevant Anatomy.

Recent data from our group has demonstrated that pituitary adenomas with low Knosp grades have the potential to invade the medial wall of the cavernous sinus, and this has been evidenced by direct visualization of the medial wall itself intraoperatively, as well as direct examination of the histological evaluation of tumor cells in the medial well itself.^1–6^

The technical nuances of resection of the medial wall of the cavernous sinus has been previously described by our group and is referenced at the end of this presentation. The relevant microsurgical anatomy will be briefly reviewed prior to presentation of the cases.^1,2,4^

Careful review of the microanatomy in this region identifies the medial wall as a single layer of meningeal dura that separates the cavernous sinus contents from the pituitary gland. The medial wall is tethered to cavernous sinus structures by important ligaments, including the carticoclinoidal ligament and the interior parasellar ligament. Equally as important in this area is identification of the inferior hypophyseal artery, which needs to be coagulated and cut for safe resection.

### 1:26 First Case Presentation.

Here we present the case of a 15-year-old girl who presented with 1 year of rapid weight gain and clear physical stigmata of Cushing’s disease. Biochemical workup confirmed the diagnosis.

A dedicated MRI of the sella revealed a hypoenhancing mass associated with the medial wall of the left cavernous sinus. Based on this imaging, it’s unclear whether this microadenoma is within the sella itself or ectopically expressed in the cavernous sinus.

Careful inspection of the lower left corner of the sellar contents did not reveal any microadenoma, despite multiple samples sent of suspicious pituitary tissue in that area; however, careful inspection of the medial wall of cavernous sinus eventually revealed the contours of a possible tumor inside the cavernous sinus, as had been suspected on the preoperative MRI.

The anterior wall of the cavernous sinus is methodically opened using a right-angled feather blade. Low-flow venous bleeding is often encountered with this maneuver, and hemostasis is achieved with light packing with Floseal. It is important that visualization is always maintained during this part of the dissection. A Doppler is used to map out the course of the carotid artery within the cavernous sinus.

### 2:32 Surgical Procedure.

A wide opening of the anterior cavernous sinus wall is essential to provide adequate visualization of the cavernous sinus contents. The inferior parasellar ligaments can frequently be encountered upon initial opening. However, when there’s significant tumor burden, as can be seen in this case, oftentimes the ligaments are poorly visualized as they can be infiltrated by tumor.

Once the tumor is visualized, circumferential dissection using bimanual technique around the tumor is methodically performed to define its surrounding dural and cavernous sinus attachments and separating it from the carotid artery.

As can be seen here, blunt and sharp dissection is used to methodically cut the dural attachments over the base of the posterior clinoid separating the medial wall of the cavernous sinus from the dural attachments of the floor of the sella.

### 3:18 Surgical Procedure and Resection of Tumor.

The final ligamentous attachments tethering the medial wall and the tumor to the roof of the cavernous sinus is a thick ligamentous structure called the caroticoclinoidal ligament, which is safely cut after being coagulated using sharp dissection.

The tumor can now be freely mobilized from the medial surface of the cavernous carotid. Careful mobilization must be performed with direct visualization of the inferior hypophyseal artery, which usually courses across the base of the posterior clinoid. The artery, as can be seen here, is intimately associated with the tumor and is coagulated and sharply cut to avoid avulsion.

The final attachments of the caroticoclinoidal ligament are sharply dissected, allowing for complete mobilization and en bloc resection of the medial wall of the cavernous sinus with the attached tumor. Final hemostasis is achieved, and direct visualization of the resection cavity is visualized with an angled endoscope. Any microscopic disease remaining on the carticoclinoidal ligament is coagulated to allow for optimal resection and maximize the opportunity for biochemical remission.

Direct examination of the surgical cavity reveals an excellent resection, and this was further supported by the patient’s cortisol nadir, which precipitously crashed over the next 24 to 48 hours.

### 4:36 Second Case Presentation.

The second case involves a 17-year-old male who presented with clinical and biochemical evidence of Cushing’s disease. He underwent two transsphenoidal surgeries with unsuccessful biochemical remission. MRI revealed residual tumor within the left cavernous sinus.

A wide sphenoidotomy and transpterygoid approach was performed to allow for the necessary lateral exposure to access the anterior cavernous sinus, as demonstrated here.

A carotid Doppler is used to methodically map out the carotid artery in relation to the anterior cavernous sinus wall.

Immediately upon opening the anterior cavernous sinus wall, the tumor is visualized.

### 5:14 Surgical Procedure.

Wide opening of the anterior cavernous sinus wall allows for complete visualization of the tumor to allow for circumferential dissection and identify the dural and ligamentous attachments that are tethering the medial wall and the tumor to the cavernous sinus contents.

The tumor is bluntly dissected off the medial surface of the carotid artery, and the final attachments of the medial wall and the tumor to the carticoclinoidal ligament are identified and sharply dissected to allow for complete mobilization.

### 5:45 Surgical Procedure and Tumor Resection.

The dural attachments along the base of the poster clinoid process are also sharply cut, untethering the medial wall from the dura of the floor of the sella. The inferior hypophyseal artery is not visualized in this case; however, careful dissection in this area and coagulation of suspicious, traversing structures were performed prior to cutting.

The tumor and medial wall are completely untethered from the cavernous sinus contents and can now be removed en bloc, resulting in gross-total resection and excellent biochemical evidence of a postoperative cortisol crash suggestive of durable biochemical remission.

### 6:19 Postoperative Course.

After nearly 2 years of follow-up, both patients remain in remission without any neurological complications or pituitary axis deficits. These two cases underscore the power of this surgical technique in achieving biochemical remission in challenging cases that either present with ectopic adenoma presentation in the cavernous sinus or in adenomas that invade the cavernous sinus.

### 6:40 Conclusions.

We recommend that such complex cases should be performed by experienced and trained surgeons, as expanded endoscopic approaches to the cavernous sinus have a steep learning curve.

## Disclosures

Dr. Fernandez-Miranda reported nonfinancial support from Stryker and personal fees from KLS Martin and Hotry, outside the submitted work.

## Author Contributions

Primary surgeon: Fernandez-Miranda. Assistant surgeon: Mohyeldin. Editing and drafting the video and abstract: Mohyeldin, Nunez, Xu. Critically revising the work: Mohyeldin, Xu, Fernandez-Miranda. Reviewed submitted version of the work: Mohyeldin, Xu, Fernandez-Miranda. Approved the final version of the work on behalf of all authors: Mohyeldin. Supervision: Mohyeldin, Fernandez-Miranda.

## Supplemental Information

### Online-Only Content

Supplemental material is available online.

*Supplemental Figs. 1 and 2*. https://thejns.org/doi/suppl/10.3171/2023.4.FOCVID2323.

### Patient Informed Consent

The necessary patient informed consent was obtained in this study.

## References

[1] Mohyeldin A Katznelson LJ Hoffman AR Prospective intraoperative and histologic evaluation of cavernous sinus medial wall invasion by pituitary adenomas and its implications for acromegaly remission outcomes Sci Rep 2022 12 1 9919 35705579 10.1038/s41598-022-12980-1 PMC9200976

[2] Truong HQ Lieber S Najera E Alves-Belo JT Gardner PA Fernandez-Miranda JC The medial wall of the cavernous sinus. Part 1: Surgical anatomy, ligaments, and surgical technique for its mobilization and/or resection J Neurosurg 2018 131 1 122 130 30192192 10.3171/2018.3.JNS18596

[3] Cohen-Cohen S Gardner PA Alves-Belo JT The medial wall of the cavernous sinus. Part 2: Selective medial wall resection in 50 pituitary adenoma patients J Neurosurg 2018 131 1 131 140 30192191 10.3171/2018.5.JNS18595

[4] Yasuda A Campero A Martins C Rhoton AL Jr Ribas GC The medial wall of the cavernous sinus: microsurgical anatomy Neurosurgery 2004 55 1 179 190 15214988 10.1227/01.neu.0000126953.59406.77

[5] Nishioka H Fukuhara N Horiguchi K Yamada S Aggressive transsphenoidal resection of tumors invading the cavernous sinus in patients with acromegaly: predictive factors, strategies, and outcomes J Neurosurg 2014 121 3 505 510 25014437 10.3171/2014.3.JNS132214

[6] Oldfield EH Cushing’s disease: lessons learned from 1500 cases Neurosurgery 2017 64 CN suppl 1 27 36 28899067 10.1093/neuros/nyx378

